# Dietary probiotics have different effects on the composition of fecal microbiota in farmed raccoon dog (*Nyctereutes procyonoides*) and silver fox (*Vulpes vulpes fulva*)

**DOI:** 10.1186/s12866-019-1491-x

**Published:** 2019-05-24

**Authors:** Yongjia Peng, Qiumei Shi, Yujie Wang, Fan Zhang, Zhixin Ji, Jin Zhang

**Affiliations:** 1 College of Biological, Chemical Sciences and Engineering, Jiaxing University, 118 Jiahang Road, Jiaxing, 314001 People’s Republic of China; 2 Hebei Normal University of Science and Technology, 360 Hebei Street, Qin Huangdao, 066004 People’s Republic of China

**Keywords:** Mixed probiotic preparation, Fox, Raccoon dog, 16S rRNA

## Abstract

**Background:**

The abuse of antibiotics in animal husbandry imposes a serious threat to both animal health and the environment. As a replacement for antibiotics, probiotic products have been widely used in livestock farming to promote growth of animals. However, no products specifically developed for farmed raccoon dogs and foxes are commercially available at the moment. This study was conducted to investigate the effects of mixed probiotics on farmed raccoon dogs and foxes.

**Results:**

Two feeding trials on farmed raccoon dogs and foxes were performed. A mixed probiotic preparation composed of *Bifidobacterium bifidum*, *Clostridium butyricum*, *Bacillus subtilis* and *Bacillus licheniformis* was fed to these two canine species in order to assess whether such a mixed probiotics can be an alternative to antibiotics (control group). The body weight of raccoon dogs exhibited an increasing tendency with mixed probiotics administration, while that of foxes did not. The serum antioxidant activity was evaluated, and a significantly increase of total antioxidative capacity (T-AOC) was observed in both species. Illumina MiSeq was used for the sequencing of 16S rRNA genes to compare the composition of fecal microbiota between the control and mixed probiotics groups. Although α-diversity did not change, β-diversity of the fecal microbiota showed a distinct dissimilarity between the control and probiotics groups of both raccoon dogs and foxes. Dietary mixed probiotics increased the abundance of the genus *Bifidobacterium* in the fecal samples of raccoon dogs, and the genus *Bacillus* in the fecal samples of foxes. The different responses of raccoon dogs and foxes to probiotics might be the result of differences in the composition of the native gut microbiota of the two species.

**Conclusions:**

The mixed probiotics preparation composed of *Bifidobacterium bifidum*, *Clostridium butyricum*, *Bacillus subtilis* and *Bacillus licheniformis* could be an effective feed additive for the improvement of the health of farmed raccoon dogs, but it may not be suitable for foxes.

**Electronic supplementary material:**

The online version of this article (10.1186/s12866-019-1491-x) contains supplementary material, which is available to authorized users.

## Background

The abuse of antibiotics in domestic animal feeding is a serious problem for both animal health and the environment [1]. Probiotic products are considered to be better feed additives and have been widely used in livestock farming [2]. Studies show that probiotics have many positive effects on host animals, including improvement of their immune system [3], reduction of stress levels [4], protection against infections [5], enhancement of growth performance, and even prevention of metabolic diseases [6, 7]. It is now widely accepted that not only specific probiotic products are needed for each kind of domestic animal species, different products should be administered for every developmental stages of each farmed animal species.

Farmed raccoon dogs and foxes, which belong to the family Canidae, are two main sources of fur. Viruses, parasites, and other pathogens are always present in wild and farmed raccoon dogs and foxes [8, 9]. However, no probiotic products specifically designed for these two species are commercially available at the moment. Colistin sulfate and zinc bacitracin are routinely added by farmers into the feed to protect the animals from pathogens [10, 11]. It is proven that the use of antibiotics decreases the death rate of animals, but the unnecessarily high dosages used in practice can also make the animals sub-healthy. At the same time, the overuse of antibiotics leads to high residues in the animals’ feces, causing soil and water pollution around the farm [12, 13]. Therefore, safer and more environmentally-friendly alternatives to the antibiotics used in raccoon dog and fox farming are urgently needed. In order to investigate the composition of the gut microbiota of these two canids, we used 16S rRNA sequencing technology to study the fecal microbiome (unpublished data). With a combination of high-throughput sequencing and relative functional studies, gut colonizers of the hosts can be characterized and their phylogenetic diversity identified, opening a promising avenue of “smart probiotics” in industrial application [14]. Thus, it is meaningful to use deep sequencing to investigate the response of gut microbiota to the administration of probiotics. In this study, we administered a patented mixture of probiotics developed by our group [Patent no. 201410586164.7], composed of *Bifidobacterium bifidum*, *Clostridium butyricum*, *Bacillus subtilis* and *Bacillus licheniformis*, to the two canids. The objective of this study was to assess whether this mixture of probiotics can be adopted as an alternative to antibiotics in the protection of farmed canids from disease.

## Results

### Dietary administration of the probiotic mixture improved the health performance of raccoon dogs

**Fig. 1:**
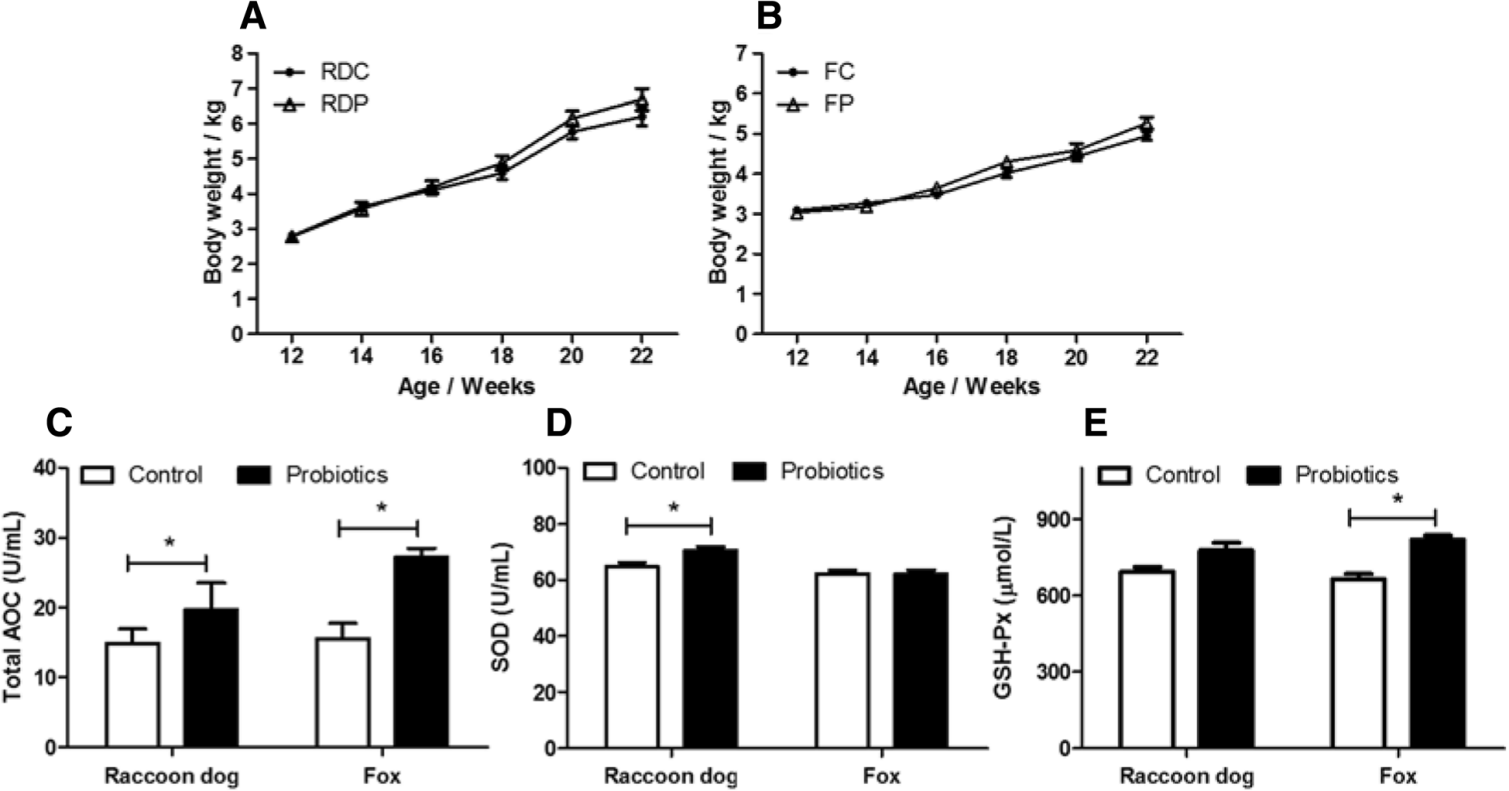
The effect of dietary probiotics on the health performance of farmed raccoon dogs and foxes. (**a**): Body weight of raccoon dogs (*n* = 32 for each group). (**b**): Body weight of foxes (*n* = 32 for each group). (**c**): Total antioxidative capacity (T-AOC) (*n* = 8 for each group of raccoon dogs, *n* = 6 for each group of foxes). (**d**): Superoxide dismutase (SOD) activity (n = 8 for each group of raccoon dogs, *n* = 6 for each group of foxes). (**e**): Glutathione peroxidase (GSH-Px) activity (n = 8 for each group of raccoon dogs, *n* = 6 for each group of foxes)

In order to evaluate the effects of the probiotic mixture on the health of the investigated farmed canids, the body weight and serum antioxidant activity were analyzed (Fig. 1). Both species were fed with the probiotic mixture from 12 to 24 weeks of age. The raccoon dogs fed with the probiotic mixture showed a tendency of higher body weight than the control group from 18 weeks old to the end, although this difference was not statistically significant (*p* = 0.072) (Fig. 1a). The body weight of the foxes fed with the probiotic mixture did not show an increasing trend (Fig. 1b).

The serum antioxidant activity was measured in the control and probiotic treatment groups. A significant increase of total antioxidative capacity (T-AOC) was observed in both raccoon dogs and foxes (Fig. 1c). Specifically, that of while superoxide dismutase (SOD) increased in only raccoon dogs (Fig. 1d), while the glutathione peroxidase (GSH-Px) one only increased in foxes (Fig. 1e).

### Dietary administration of the probiotic mixture altered the fecal bacterial community compositions of raccoon dogs and foxes

The fecal samples of raccoon dogs and foxes treated with/without probiotics were analyzed using lllumina high-throughput sequencing technology to investigate the diversity of microbial communities. The total averages of OTUs and taxonomic information were identified at the 97% sequence similarity level in each sample. In the raccoon dog samples, 743 OTUs resulted in 20 phyla and 229 genera in the control group, while 1659 OTUs resulted in 30 phyla and 361 genera in the probiotics group. In the fox samples, 462 OTUs resulted in 6 phyla and 70 genera in the control group and 454 OTUs resulted in 8 phyla and 75 genera in the probiotics group (Additional file 1: Table S1).

**Fig. 2:**
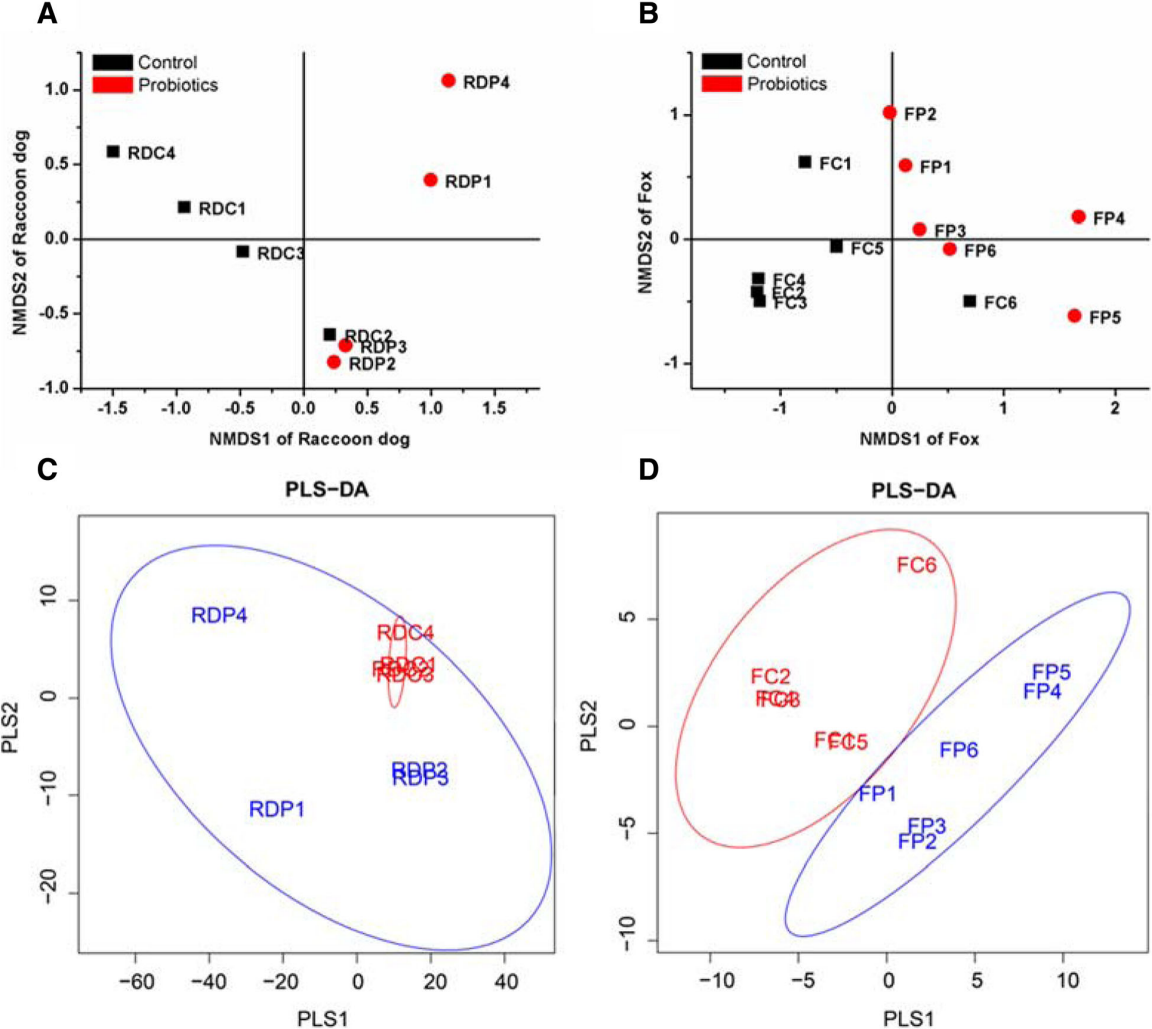
Nonmetric multidimensional scaling (NMDS) ordination with weighted UniFrac distances of the control and probiotic treatment groups. Solid black squares represent control samples, and solid red circles represent probiotic-treated samples. (**a**): NMDS plot of raccoon dog control and probiotic groups. (**b**): NMDS plot of fox control and probiotic groups. Partial least squares discriminant analysis (PLS-DA) based on a supervised model was used to reveal the variation of microbiota between the control and probiotic groups; red circles represent control samples, blue circles represent probiotic samples. (**c**): PLS-DA plot of raccoon dog control and probiotic groups. (**d**) PLS-DA plot of fox control and probiotic groups

Differences of α-diversity of microbiota between the samples from the control and mixed probiotics administered groups were evaluated, and neither diversity (Shannon and Simpson) nor richness estimators (Chao1 and ACE) exhibited significant differences in both species when treated with mixed probiotics (Additional file 2: Table S2). However, the NMDS plot based on weighted UniFrac distances revealed a dissimilarity between the control and probiotic treatment groups (Fig. 2a and b). Both species showed a similar trend to that of the control group, except for one individual positioned in the left part (second and third quadrants), and all probiotic groups in the right part (first and forth quadrants). Partial least squares-discriminant analysis (PLS-DA) showed similar results, with individuals treated with probiotics clustering together, with a clear demarcation from the control groups in both species (Fig. 2c and d).

### Dietary administration of the mixed probiotic preparation affected the composition of fecal microbiota in the raccoon dogs and foxes differently

**Fig. 3:**
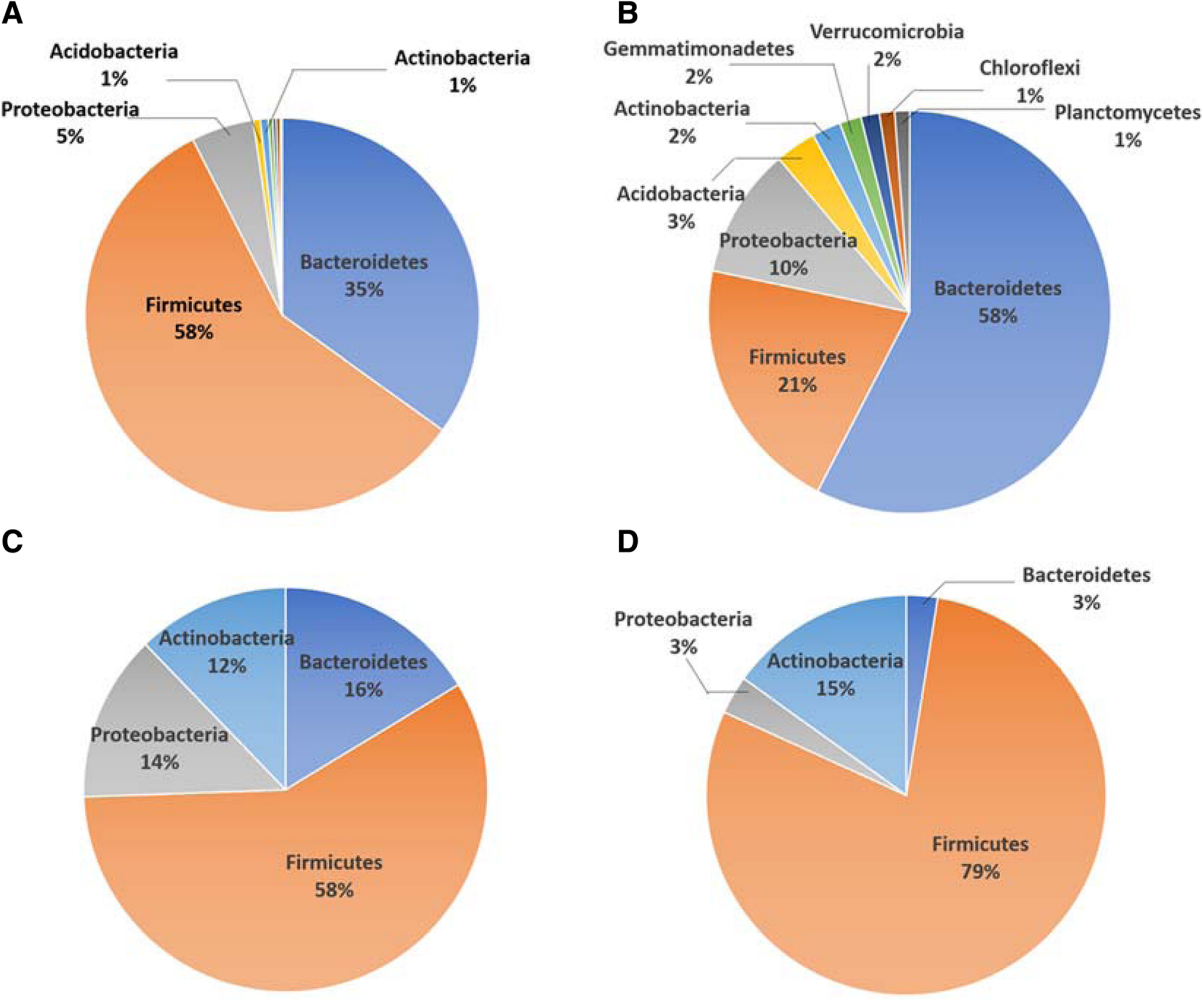
Distribution of the fecal microbiota at the phylum level. (**a**): raccoon dog control group. (**b**): raccoon dog probiotic group. (**c**): fox control group. (**d**): fox probiotic group

The composition of microbiota was analyzed at the phylum and genus levels to unveil the effect of the mixed probiotics. In the RDC group, a total of 16 phyla were identified and five phyla (Bacteroidetes, Firmicutes, Proteobacteria, Acidobacteria and Actinobacteria) remained if the threshold was set over 1% of total microbiota (Fig. 3a). In the RDP group, 19 phyla were identified and nine phyla remained if the threshold was set over 1% of total microbiota (Fig. 3b). Although the top five phyla were the same in the RDC and RDP groups, the ratio of each phylum changed significantly. Firmicutes decreased, while Bacteroidetes, Proteobacteria, Acidobacteria and Actinobacteria increased with administration of the mixed probiotics. Overall, the microbiota of raccoon dogs tended to be more diverse, even with the administration of mixed probiotics. In the fox fecal microbiota, a smaller number of phyla was identified, so that Firmicutes, Bacteroidetes, Proteobacteria and Actinobacteria accounted for over 99% of total microbiota. When treated with the probiotics, Firmicutes increased, while Bacteroidetes and Proteobacteria decreased in the fecal microbiota of the foxes (Fig. 3c and d). In the FC group, a total of 7 phyla were identified, and four phyla (Bacteroidetes, Firmicutes, Proteobacteria, and Actinobacteria) remained if the threshold was set over 1% of total microbiota (Fig. 3c). In the FP group, 9 phyla were identified and four phyla remained if the threshold was set over 1% of total microbiota (Fig. 3d). Although the top four phyla were the same in the FC and FP groups, the ratio of each phylum changed significantly. Firmicutes increased, while Bacteroidetes and Proteobacteria decreased upon administration of the mixed probiotics. Surprisingly, the microbiota of the foxes did not show an increased diversity with the administration of mixed probiotics.

**Fig. 4:**
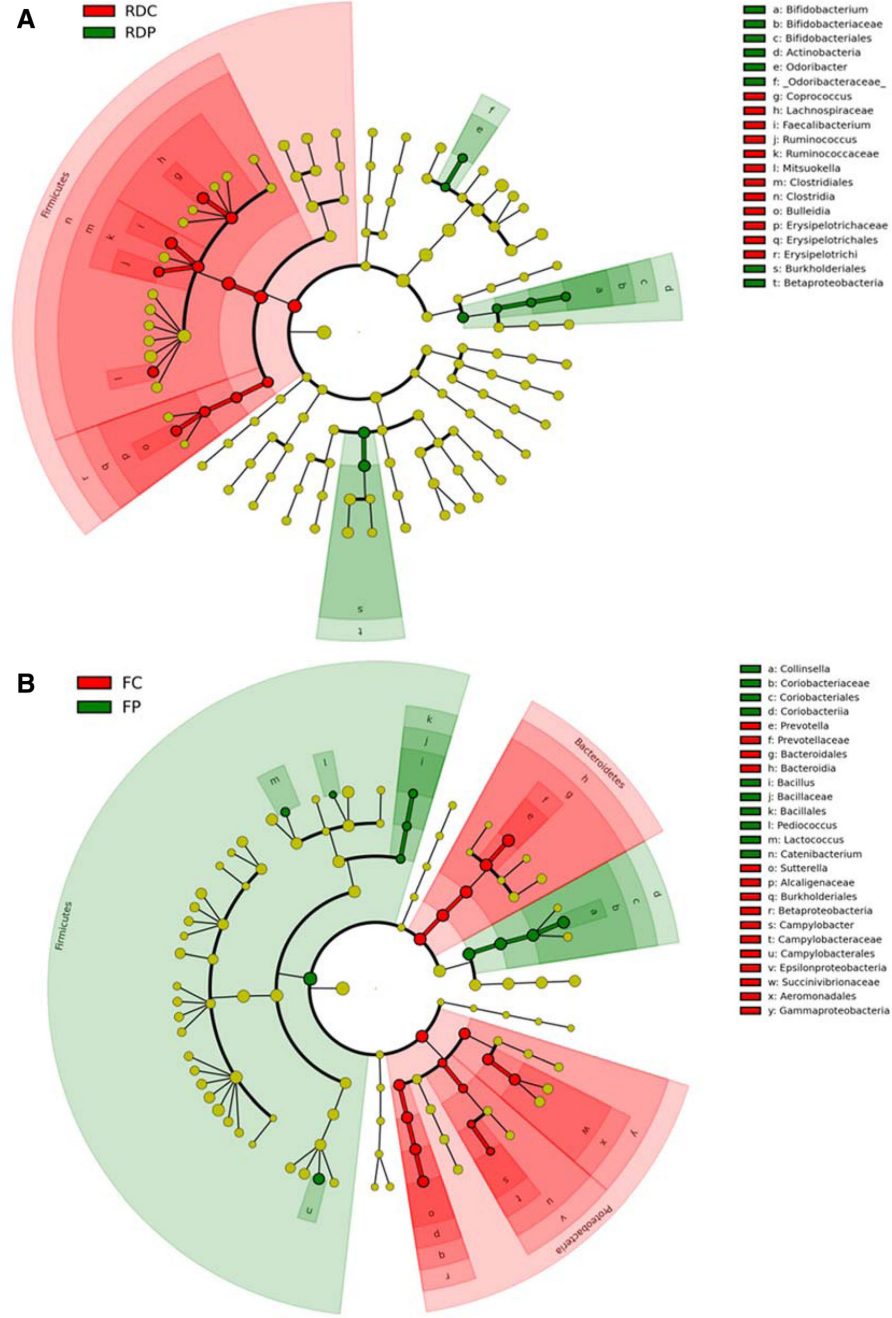
Cladogram plotted from the data of the Linear discriminant analysis and effect size (LEfSe) analysis of the probiotic treatment and control groups. The green circles represent clades with significantly higher abundance in the probiotic treatment groups, red circles represent clades with significantly higher abundance in the control groups, and yellow circles represent microbial clades without significant differences of abundance between the groups. Concentric rings from inside to the outside denote the taxonomical levels of phylum, class, order, family and genus. (**a**): Differentially abundant microbial clades in the fecal samples of raccoon dogs from the group that underwent probiotic treatment (RDP, *n* = 4) versus control (RDC, n = 4). (**b**): Differentially abundant microbial clades in the fecal samples from foxes that underwent probiotic treatment (FP, *n* = 6) versus control (FC, *n* = 6)

To further identify the specific fecal microbiota which significantly changed in response to probiotic treatment, Linear discriminant analysis and effect size (LEfSe) analysis were used to identify the specific taxonomic differences between the control and probiotic groups. In the raccoon dog trial, probiotic treatment increased the proportion of the genus *Bifidobacterium*, which belongs to the class Actinobacteria, order Bifidobacteria, and family Bifidobacteriaceae (Fig. 4a and Additional file 4: Figure S2A). Additionally, the genus *Odoribacter* from the family Odoribacteraceae, and order Burkholderiales from the class Betaproteobacteria was also higher in the probiotics group. On the other hand, a higher relative percentage of the phylum Firmicutes, including the genera *Coprococcus*, *Faecalibacterium*, *Ruminococcus*, *Bulleidia* and *Mitsuokella* was detected in fecal microbiota from the raccoon dog control group. Interestingly, the effect of probiotic treatment on the composition of fecal microbiota in the fox trial was different from what was observed in the raccoon dog trial. The genus *Bacillus* from the family Bacillaceae, order Bacillales, phylum Firmicutes, and the genus *Collinsella* from the family Coriobacteriaceae, order Coriobacteriales, subclass Coriobacteridae within the class Actinobacteria were enriched in the probiotic treatment group (Additional file 5: Figure S2B). In contrast, larger abundances of the genera *Prevotella*, *Sutterella*, and *Campylobacter*, as well as the family Succinivibrionaceae, were found in the fox control group (Fig. 4b). Compared with each control group, a lower abundance of the genus *Bulleidia* was detected in the RDP group (Additional file 5: Figure S2C), as well as the genera *Campylobacter* and *Sutterella* in the FP group (Additional file 5: Figure S2D and E), all of which are potential pathogens.

**Fig. 5:**
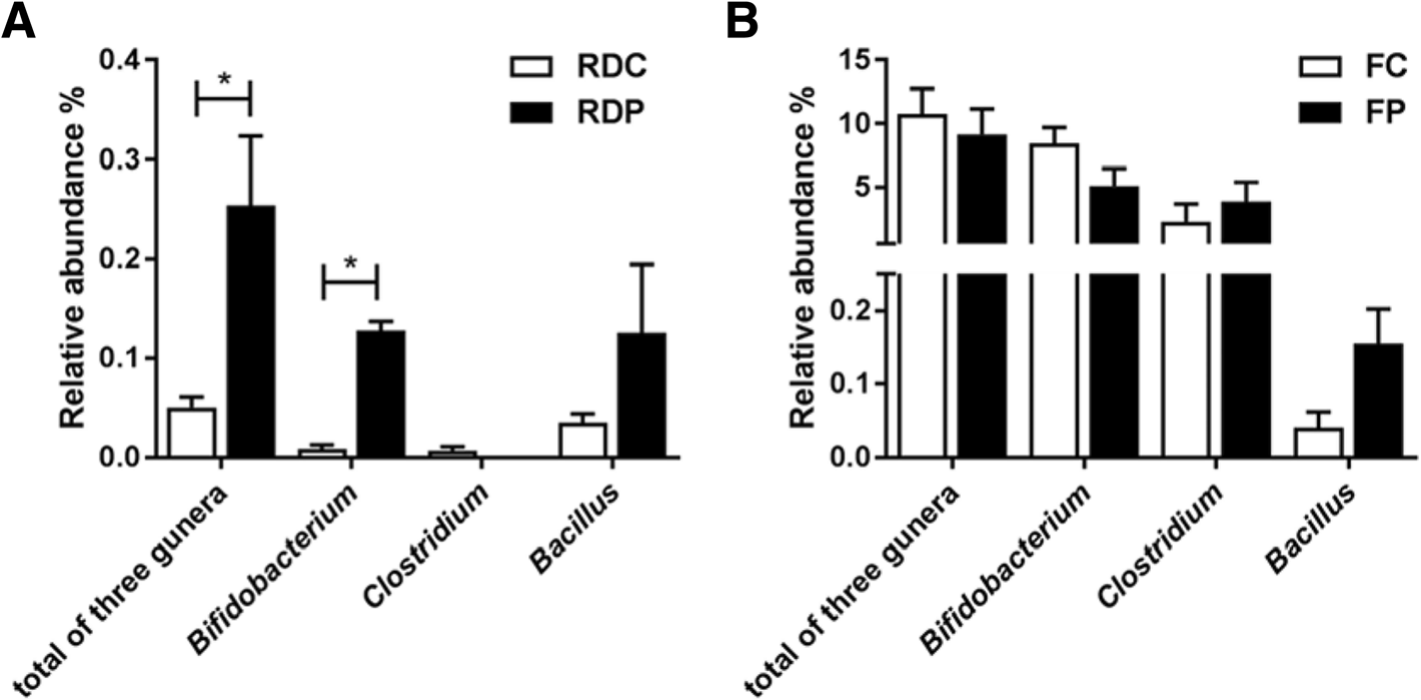
Relative abundance of the genera added to the probiotic mixture. (**a**): Raccoon dog control and probiotic group. (**b**): Fox control and probiotic group

In order to evaluate the colonization and proliferation of the probiotics, the relative abundance of the four stains (*Bifidobacterium bifidum*, *Clostridium butyricum*, *Bacillus subtilis* and *Bacillus licheniformis*) in the canine fecal samples was analyzed at the genus level. The relative abundance of the genus *Bifidobacterium* was significantly higher in the RDP samples than in the RDC samples, and the total amount of the three genera in the RDP samples increased almost 5 times compared to the RDC samples (Fig. 5a). However, no difference was found in individual or total genera between the fox samples. Only the relative abundance of the genus *Bacillus* showed an increasing trend, which was not statistically significant (*p* = 0.061) (Fig. 5b).

## Discussion

According to experiences from conventional animal husbandry, the supplementation of antibiotics in the feed has two positive effects. One is protecting the animals from diarrhea during the season transitions, which may cause animal death, and the other is promoting animal growth. Since the potential negative effects of antibiotics overuse on animal health and the environment have become apparent, probiotics are increasingly being considered as an alternative in farming and medicine [15]. However, there is yet no consensus regarding the therapeutic efficacy of probiotics [16]. In this study, we investigated the effects of a mixed probiotic preparation on the health performance of farmed raccoon dogs and foxes. With the aim of developing an alternative to antibiotics for different farmed fur animals, antibiotic-treated animals were used as control in both raccoon dog and fox trials. In this case, whether the effects of the same probiotics on raccoon dogs and foxes were similar or not could be identified. During the entire experimental process, no deaths were recorded in either of the two species. While the growth promotion effect was observed in raccoon dogs, it was absent in foxes. The results thus clearly show that the mixed probiotics exhibited a good efficacy on the raccoon dogs, but had no obviously better effect on the foxes compared with antibiotics. Furthermore, none-antibiotic-treated animals were not included in this study, due to two reasons. First of all, the purpose of this study is to find a replacement for the antibiotics. Secondly, the farm conditions did not allow the rearing of unprotected animals. Raccoon dogs or foxes raised on a farm without either antibiotics or probiotics are at high risk of being infected by pathogens, which may create an epidemic on the farm, and hence represents an unacceptable hazard. Based on these two considerations, the fact that none-antibiotic-treated animals were not involved in this study needs to be considered when this study is used to be compared with others.

Serum antioxidative activity, which can indicate host health status, was measured to evaluate the effects of the mixed probiotics on overall health. As expected, both raccoon dogs and foxes in the probiotics group showed a higher serum antioxidative activity than the ones in the antibiotics group. Effects of probiotics on antioxidative activity have been observed in humans and rodents [17, 18]. *Bifidobacterium bifidum*, one component of the mixed probiotics, was reported to have the function of protecting against oxidative stress and decreasing the accumulation of reactive oxygen metabolites [17]. Not surprisingly, the probiotic mixture that included *Bifidobacterium bifidum* displayed an enhanced serum antioxidant activity in both raccoon dogs and foxes and this mixture has a positive effect on the overall health of the two canids.

In order to understand why the two canids responded differently to the same probiotic product, we analyzed the composition of fecal microbiota via high-throughput 16S rDNA sequencing. In samples from both raccoon dogs and foxes, α-diversity did not show a significant difference after probiotic administration. Nevertheless, the β-diversity showed a clear dissimilarity between the control and probiotic groups. Moreover, PLA-DA also showed a consistent trend of β-diversity. These data suggested that the mixed probiotics altered the gut microbiota of the two canid species in a subtle way. Several previous reports found similar positive effects of the addition of probiotic feed to non-diseased dogs, cats and farmed livestock [6, 19].

In the raccoon dog trial, the feeding of mixed probiotics produced a more diverse and even composition of bacterial phyla. The top two phyla, Firmicutes and Bacteroidetes, accounted for 93% in the RDC group and 79% in the RDP group. The decrease of the dominant phyla was accompanied with an increase of other phyla (Fig. 3b), which implies a positive role of the mixed probiotics treatment on intestinal biodiversity [20]. In terms of the composition of fecal microbiota at deeper taxonomical ranks, a major change within the phylum Actinobacteria was observed, with an increase of the genus *Bifidobacterium* in the RDP group. Bifidobacteria are well-known inhabitants of the intestinal tract, which play an important role in establishing a balance in the gut microbiota and thus provide health benefits to the host [21]. Notably, the relative abundance of the genus *Bulleidia*, which is potentially associated with infectious diseases [22], decreased significantly with the supplementation of probiotics (Fig. 4a and Additional file 5: Figure S2C). The alteration of the microbiota may be the proximal cause for the observed health improvement of the raccoon dogs produced by the mixed probiotic supplement in the diet.

In the fox trial, the Firmicutes phylum accounted for 58% in the FC group and 79% in the FP group. The increase of Firmicutes was accompanied with a decrease of other phyla (Fig. 3d). Different from the raccoon dog trial, the administration of mixed probiotics did not induce a more diverse and even composition of bacterial phyla in the foxes. A further investigation of the composition at deeper taxonomic levels revealed that the probiotic supplementation increased the abundance of the genus *Bacillus*, which belongs to the phylum Firmicutes. On the other hand, several genera were less abundant in the FP samples, such as the genus *Sutterella* and genus *Campylobacter*, which are potentially related to inflammation and infections [23, 24]. Since antibiotic treatment can lead to an increased abundance of *Sutterella* [25], it appears that the probiotic mix might also suppress the harmful microbes in the fox gut, circumventing the potential negative effects of antibiotics on gut microbiota.

The mixed probiotic was composed of *Bifidobacterium bifidum*, *Clostridium butyricum*, *Bacillus subtilis* and *Bacillus licheniformis*. We analyzed the relative amounts of these species in the two canids’ fecal samples at the genus level. As discussed previously, the abundance of the genus *Bifidobacterium* increased in the fecal samples of raccoon dogs, while the genus *Bacillus* increased in the fecal samples of foxes that were administered with the mixed probiotics. This suggests that the dietary probiotics colonized and proliferated in the hosts’ intestines. Interestingly, the colonization and proliferation of the four species was quite different in raccoon dogs and foxes. In the raccoon dog control group, we found that the relative amounts of the four stains were very low (lower than 0.5%), which was especially true for *Bifidobacterium*. In contrast, these species were relatively abundant in foxes (more than 4%), except for *Bacillus* (0.4%) (Fig. 5). These results may explain why the two canids responded differently to the same mixed probiotics. It is likely that dietary probiotics increase specific microbes in a host-dependent manner, which implies that specific probiotic products should be developed for each farmed animal, or even each growth stage.

## Conclusions

The mixed probiotic preparation composed of *Bifidobacterium bifidum*, *Clostridium butyricum*, *Bacillus subtilis* and *Bacillus licheniformis* could be an effective feed additive for the improvement of the health of farmed raccoon dogs, but is not suitable for foxes in this form.

## Methods

### Ethical approval

The experimental procedures used here, including the feeding, transport and slaughter of the subject raccoon dogs and foxes, were approved by the Jiaxing University Experimental Animal Welfare Ethics Committee.

## Preparation of probiotics

A mixed culture of *Bifidobacterium bifidum* (CGMCC 1.5091), *Clostridium butyricum* (CGMCC 1.336), *Bacillus subtilis* (CGMCC 1.1731) and *Bacillus licheniformis* (CGMCC 1.813) was obtained on solid state medium, as described in our previous study [26]. The number of live bacteria in the mixed probiotics was 1 × 10^12^ CFU/g.

### Animals and sample collection

Two feeding experiments were conducted separately, one on raccoon dogs, (*Nyctereutes procyonoides*) and the other on foxes (*Vulpes vulpes fulva*). Raccoon dogs and foxes were bred and raised in the farm of Hua Xia Xin Nong technology co., LTD, Hebei province, China (E119.15, N39.72) from 12 until 22 weeks of age. Each animal was caged individually without temperature/humidity control. All the animals were fed with a basal diet (Additional file 3: Table S3) twice daily and given free access to drinking water.

In the raccoon dog trial, sixty-four raccoon dogs weighing 2.79 ± 0.35 kg were randomly divided into two groups, with each group composed of thirty-two individuals. One group was raised in accordance with commercial husbandry, and was named as the control group (RDC). In this group, the feed was supplemented with antibiotics (20 ppm colistin sulfate and 100 ppm zinc bacitracin, Shandong Guobang Pharmaceutical Company LTD, China). The other group was named as the probiotic group (RDP), whose feed was supplemented with the probiotics mixture (0.1% dry matter) instead of the antibiotics. The body weight was recorded every two weeks. At the end of the trial, blood samples were taken from eight randomly chosen individuals, and fresh fecal samples were collected from four racoon dogs in each group (Additional file 4: Figure S1A).

In the fox trial, sixty foxes weighing 3.06 ± 0.17 kg were randomly divided into two groups, with each group composed of thirty individuals. The arrangement was similar to the raccoon dog trial. One group was raised according to commercial husbandry and was named as the control group (FC). In this group, the feed was supplemented with antibiotics (20 ppm colistin sulfate and 100 ppm zinc bacitracin, Shandong Guobang Pharmaceutical Company LTD, China). The other group was named as the probiotics group (FP), whose feed was supplemented with the probiotics mixture (0.1% dry matter) instead of the antibiotics. The body weight was recorded every two weeks. At the end of the trial, blood and fresh feces samples were respectively collected from two batches of six randomly-chosen individuals in each group. When the feeding trials were finished, all animals were fed for another 3 or 4 more months and were sacrificed by electroshock for fur production.

### Analysis of serum antioxidant activity

Serum total antioxidative capacity (T-AOC), superoxide dismutase (SOD) and glutathione peroxidase (GSH-Px) were analyzed using commercial kits from Nanjing Jiancheng Bioengineering Institute, China. The serum activities of T-AOC and SOD were expressed as units per milliliter, and the serum activity of GSH-Px was expressed in micromoles per liter.

### 16S rRNA analysis of the fecal bacteria

Microbial genomic DNA was extracted from fecal samples using the TIANGEN DNA stool mini kit (DP328, TIANGEN, China). The primers 520F (5′-AYTGGGYDTAAAGNG-3′) and 802R (5′-TACNVGGGTATCTAATCC-3′) specific for 16S rRNA gene were used to amplify the V4 region [27]. The PCR products were pooled and purified using the QIAGEN quick Gel Extraction Kit (28706, QIAGEN, Germany). The DNA library was constructed and run on the Illumina MiSeq platform at Shanghai Personal Biotechnology Co., Ltd. (Shanghai, China). The raw reads were deposited into the European Nucleotide Archive database under the accession code PRJEB24008, and the NCBI sequence reads archive (SRA) under the accession number SRA641236.

### Sequence analysis

To obtain valid sequences, raw data were assembled using Flash (v1.2.7, http://ccb.jhu.edu/software/FLASH/), following quality filtering and data analysis in QIIME (v1.8.0, http://qiime.org/) [28, 29]. The library sizes of the raccoon dog and fox samples were rarefied to a depth of 26,000 and 65,000 clean reads, respectively. The OTUs (operational taxonomic units) were identified using the UCLUST Algorithm (http://drive5.com/usearch/manual/uclust_algo.html) in QIIME with an identity threshold of 97%. The representative sequences were chosen and blasted against the GreenGenes database (Release 13.8, http://greengenes.secondgenome.com/). The α-diversity within the samples was calculated using QIIME http://qiime.org/scripts/ alpha_diversity.html). Four indices were included: Chao1, Ace, Shannon diversity and Simpson. β-diversity was obtained by calculating the weighted and unweighted UniFrac distances, and NMDS (non-metric multidimensional scaling) plots were generated based on weighted UniFrac distances. PLS-DA (partial least squares discriminant analysis) was also introduced as a supervised model to reveal the variation of microbiota among groups, using the “plsda” function in R package “mixOmics” [30]. The analysis of LDA effect sizes (LEfSe) was conducted for the quantitative analysis of biomarkers between control and probiotics treatment groups, and this process was performed online in the Galaxy workflow framework (https://huttenhower.sph.harvard.edu/galaxy/) [31].

### Statistical analysis

The comparisons of body weight, serum T-AOC, SOD, GSH-Px, the Shannon, Simpson, Chao1 and ACE α-diversity indices, and relative abundances of probiotic genera between the control and probiotic-administration groups were performed using the Wilcoxon *t*-test. Differences with *p* < 0.05 were considered statistically significant. The data were presented as the means ± SE. The software package SPSS (v19.0, IBM, Armonk, NY, USA) was used to perform the statistical analyses.

Linear discriminant analysis (LDA) and effect size (LEfSe) analysis were performed to identify differentially abundant operational taxonomic units between the control and probiotic groups. The LDA threshold > 2, nonparametric factorial Kruskal-Wallis sum-rank test and Wilcoxon rank-sum test were used to identify the most differently abundant taxa, and a *p-*value < 0.05 was considered to indicate significance for all taxa.

## Additional files

Additional file 1: **Table S1.** Number of sequences analyzed. (DOCX 16 kb)

Additional file 2: **Table S2**. The alpha diversity indices of fecal microbiota of animals subjected to probiotic treatment (Mean ± SD). (DOCX 14 kb)

Additional file 3: **Table S3.** Composition and nutrient levels of the experimental diet (dry matter basis). (DOCX 14 kb)

Additional file 4: **Figure S1.** The arrangement of animal feeding trials. (A): In raccoon dog trial, sixty-four raccoon dogs were randomly divided into two groups. Each animal was caged individually without temperature/humidity control. There were four rows of cages. In control group, two individuals from each row were randomly selected for blood analysis and one individual from each row were randomly selected for fecal analysis. The sampling for probiotics group was exactly the same to the control group. (B): In fox trial, sixty foxes were randomly divided into two groups. Each animal was caged individually in yard without temperature/humidity control. There were three rows of cages. For control group, two individuals from each row were randomly selected for blood analysis and two individuals from each row were randomly selected for fecal analysis. The sampling for probiotics group was exactly the same to the control group. (PDF 118 kb)

Additional file 5: **Figure S2.** Relative abundance of bacteria in the farmed animals from the control and probiotic administration groups. (A): The LEfSe method revealed a significant difference in the relative abundance of *Bifidobacterium* between the raccoon dog control and probiotic treatment groups. (B): The LEfSe method revealed a significant difference in the relative abundance of *Bacillus* between the fox control and probiotic treatment groups. (C): The LEfSe method revealed a significant difference in the relative abundance of *Bulleidia* between the raccoon dog control and probiotic treatment groups. (D): The LEfSe method revealed a significant difference in the relative abundance of *Campylobacter* between the fox control and probiotic treatment groups. (E): The LEfSe method revealed a significant difference in the relative abundance of *Sutterella* between the fox control and probiotic treatment groups. (PDF 135 kb)

## Abbreviations

GSH-Px: glutathione peroxidase
LDA: Linear discriminant analysis
LEfSe: Linear discriminant analysis and effect size
NMDS: non-metric multidimensional scaling
OTUs: operational taxonomic units
PLS-DA: Partial least squares discriminant analysis
SOD: superoxide dismutase
T-AOC: total antioxidative capacity

## Authors’ information

1: College of Biological, Chemical Science and Engineering, Jiaxing University, 118 Jiahang Road, Jiaxing 314001, People’s Republic of China.

2: Hebei Normal University of Science and Technology, 360 Hebei Street, Qin Huangdao 066004, People’s Republic of China.
