# Supplementary material for: Dietary probiotics have different effects on the composition of fecal microbiota in farmed raccoon dog (Nyctereutes procyonoides) and silver fox (Vulpes vulpes fulva)
Source: BMC Microbiol. 2019 May 24;19:109. doi: 10.1186/s12866-019-1491-x (PMC6534910; doi:10.1186/s12866-019-1491-x)
Supplement: Supplementary file 1 — Table S1. Number of sequences analyzed. (DOCX 16 kb) [file 12866_2019_1491_MOESM1_ESM.docx]

**Table S3 Composition and nutrient levels of experimental diet (dry matter basis)**

| **Ingredients** | **Percentage** | **Nutrient level** | **Percentage** |
| --- | --- | --- | --- |
| Corn grain | 33.125 | Crude protein (CP) | 27.09 |
| Wheat bran | 10 | Metabolic energy (ME) | 12.96 |
| Soybean meal | 27.5 | Ether extract (EE) | 9.66 |
| Fish meal | 17 | Crude fiber (CF) | 2.92 |
| Poultry fat | 7 | Ash | 4.75 |
| Premix | 4 | Ca | 0.94 |
| CaHPO_4_ | 0.7 | P | 0.77 |
| Vitamins | 0.025 | Lys | 1.74 |
| Microelement | 0.2 | Met | 0.71 |
| Choline chloride | 0.05 | Cys | 0.36 |
| NaCl | 0.2 | Thr | 1.08 |
| DL-methionine | 0.2 | Trp | 0.32 |
