# Supplementary material for: Dietary probiotics have different effects on the composition of fecal microbiota in farmed raccoon dog (Nyctereutes procyonoides) and silver fox (Vulpes vulpes fulva)
Source: BMC Microbiol. 2019 May 24;19:109. doi: 10.1186/s12866-019-1491-x (PMC6534910; doi:10.1186/s12866-019-1491-x)
Supplement: Supplementary file 2 — Table S2. The alpha diversity indices of fecal microbiota of animals subjected to probiotic treatment (Mean ± SD). (DOCX 14 kb) [file 12866_2019_1491_MOESM2_ESM.docx]

**Table S1 Number of sequences analyzed.**

| **sample** | **sequence number** | **phylum** | **genus** | **OTU number** |
| --- | --- | --- | --- | --- |
| RDC1 | 29668 | 18 | 188 | 561 |
| RDC2 | 52991 | 22 | 251 | 772 |
| RDC3 | 59389 | 19 | 223 | 740 |
| RDC4 | 51308 | 22 | 253 | 898 |
| RDP1 | 131642 | 42 | 526 | 2536 |
| RDP2 | 52898 | 18 | 173 | 538 |
| RDP3 | 53123 | 17 | 167 | 501 |
| RDP4 | 140270 | 44 | 581 | 3060 |
| FC1 | 85597 | 5 | 67 | 398 |
| FC2 | 93682 | 6 | 70 | 453 |
| FC3 | 85873 | 8 | 72 | 501 |
| FC4 | 85588 | 6 | 67 | 471 |
| FC5 | 146484 | 4 | 63 | 544 |
| FC6 | 73781 | 9 | 79 | 405 |
| FP1 | 126662 | 6 | 64 | 548 |
| FP2 | 142605 | 6 | 65 | 488 |
| FP3 | 88288 | 4 | 74 | 468 |
| FP4 | 77517 | 11 | 82 | 380 |
| FP5 | 81665 | 12 | 89 | 381 |
| FP6 | 74125 | 8 | 78 | 456 |
