# Supplementary material for: Dietary probiotics have different effects on the composition of fecal microbiota in farmed raccoon dog (Nyctereutes procyonoides) and silver fox (Vulpes vulpes fulva)
Source: BMC Microbiol. 2019 May 24;19:109. doi: 10.1186/s12866-019-1491-x (PMC6534910; doi:10.1186/s12866-019-1491-x)
Supplement: Supplementary file 3 — Table S3. Composition and nutrient levels of the experimental diet (dry matter basis). (DOCX 14 kb) [file 12866_2019_1491_MOESM3_ESM.docx]

**Table S2 The alpha diversity indices of fecal microbiota of animal subject to probiotics treatment (Mean ± SD).**

|  | **Raccoon dog (n=4)** | |  | **Fox (n=6)** | |
| --- | --- | --- | --- | --- | --- |
|  | **Control** | **Probiotic** |  | **Control** | **Probiotic** |
| **Chao1** | 809.92±91.58 | 1802.63±697.73 |  | 473.19±25.53 | 462.13±30.45 |
| **ACE** | 849.73±105.00 | 1908.50±731.70 |  | 477.85±26.39 | 466.89±32.41 |
| **Simpson** | 0.89±0.02 | 0.88±0.06 |  | 0.90±0.02 | 0.90±0.01 |
| **Shannon** | 5.41±0.20 | 6.5±1.31 |  | 4.65±0.20 | 4.75±0.13 |
