# Supplementary material for: Dietary probiotics have different effects on the composition of fecal microbiota in farmed raccoon dog (Nyctereutes procyonoides) and silver fox (Vulpes vulpes fulva)
Source: BMC Microbiol. 2019 May 24;19:109. doi: 10.1186/s12866-019-1491-x (PMC6534910; doi:10.1186/s12866-019-1491-x)
Supplement: Supplementary file 4 — Figure S1. The arrangement of animal feeding trials. (A): In raccoon dog trial, sixty-four raccoon dogs were randomly divided into two groups. Each animal was caged individually without temperature/humidity control. There were four rows of cages. In control group, two individuals from each row were randomly selected for blood analysis and one individual from each row were randomly selected for fecal analysis. The sampling for probiotics group was exactly the same to the control group. (B): In fox trial, sixty foxes were randomly divided into two groups. Each animal was caged individually in yard without temperature/humidity control. There were three rows of cages. For control group, two individuals from each row were randomly selected for blood analysis and two individuals from each row were randomly selected for fecal analysis. The sampling for probiotics group was exactly the same to the control group. (PDF 118 kb) [file 12866_2019_1491_MOESM4_ESM.pdf]

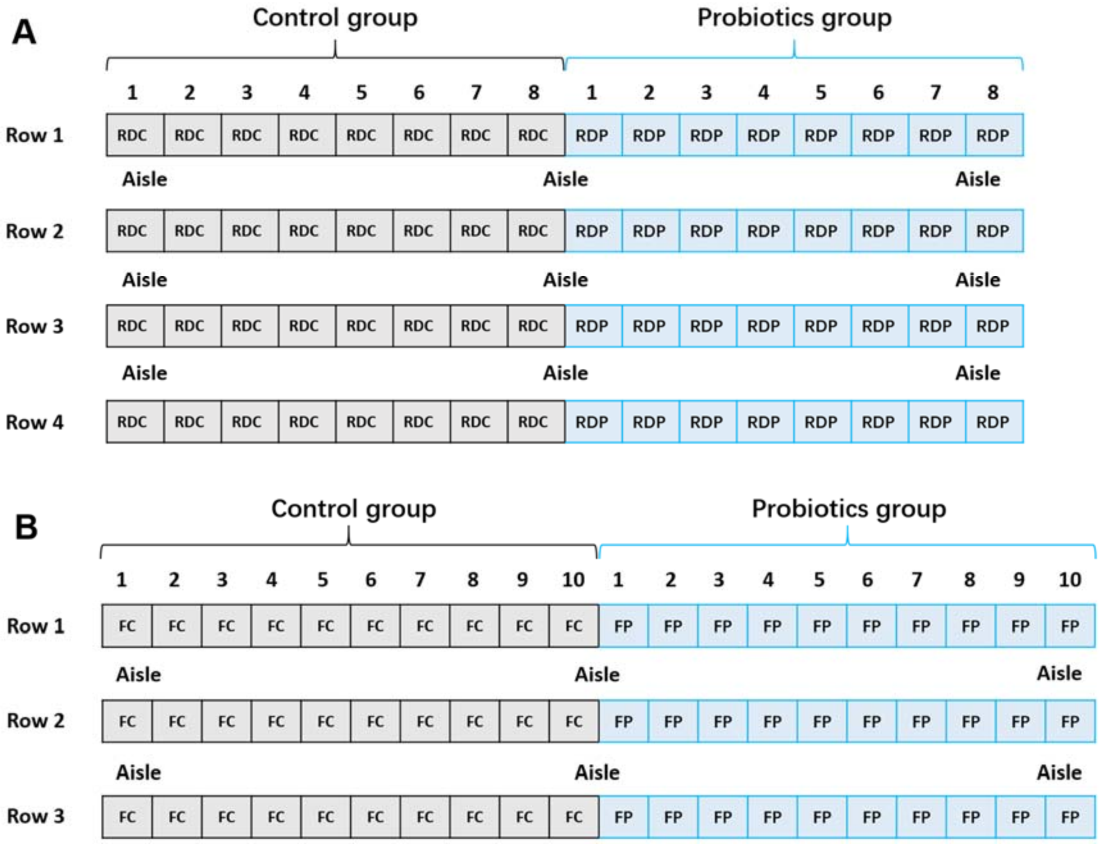
A
Control group
Probiotics group
1 2 3 4 5 6 7 8 1 2 3 4 5 6 7 8
Row 1
RDC RDC RDC RDC RDC RDC RDC RDC RDP RDP RDP RDP RDP RDP RDP RDP
Aisle Aisle Aisle
Row 2
RDC RDC RDC RDC RDC RDC RDC RDC RDP RDP RDP RDP RDP RDP RDP RDP
Aisle Aisle Aisle
Row 3
RDC RDC RDC RDC RDC RDC RDC RDC RDP RDP RDP RDP RDP RDP RDP RDP
Aisle Aisle Aisle
Row 4
RDC RDC RDC RDC RDC RDC RDC RDC RDP RDP RDP RDP RDP RDP RDP RDP
B
Control group
Probiotics group
1 2 3 4 5 6 7 8 9 10 1 2 3 4 5 6 7 8 9 10
Row 1
FC FC FC FC FC FC FC FC FC FC FP FP FP FP FP FP FP FP FP FP
Aisle Aisle Aisle
Row 2
FC FC FC FC FC FC FC FC FC FC FP FP FP FP FP FP FP FP FP FP
Aisle Aisle Aisle
Row 3
FC FC FC FC FC FC FC FC FC FC FP FP FP FP FP FP FP FP FP FP
