# Supplementary figures and images for: Dietary probiotics have different effects on the composition of fecal microbiota in farmed raccoon dog (Nyctereutes procyonoides) and silver fox (Vulpes vulpes fulva)
Source: BMC Microbiol. 2019 May 24;19:109. doi: 10.1186/s12866-019-1491-x (PMC6534910; doi:10.1186/s12866-019-1491-x)

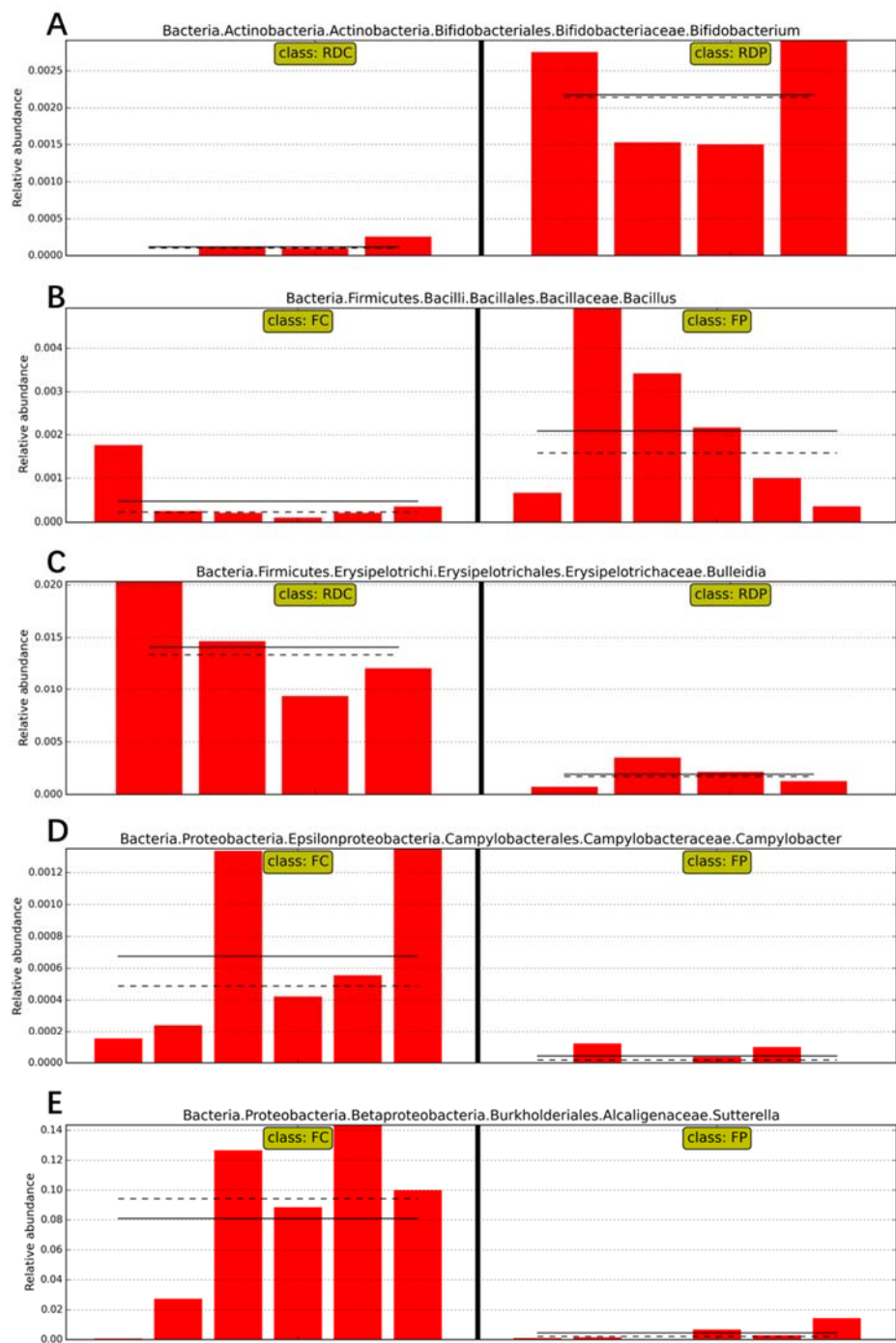

Supplement: Supplementary file 5 — Figure S2. Relative abundance of bacteria in the farmed animals from the control and probiotic administration groups. (A): The LEfSe method revealed a significant difference in the relative abundance of Bifidobacterium between the raccoon dog control and probiotic treatment groups. (B): The LEfSe method revealed a significant difference in the relative abundance of Bacillus between the fox control and probiotic treatment groups. (C): The LEfSe method revealed a significant difference in the relative abundance of Bulleidia between the raccoon dog control and probiotic treatment groups. (D): The LEfSe method revealed a significant difference in the relative abundance of Campylobacter between the fox control and probiotic treatment groups. (E): The LEfSe method revealed a significant difference in the relative abundance of Sutterella between the fox control and probiotic treatment groups. (PDF 135 kb) [file 12866_2019_1491_MOESM5_ESM.pdf]
